# Our Field, and the Two-edge Sword

**DOI:** 10.1097/GOX.0000000000004763

**Published:** 2023-01-20

**Authors:** Badr M. I. Abdulrauf

**Affiliations:** 1 From the Section of Plastic Surgery, Department of Surgery, King Faisal Specialist Hospital and Research Center (General Organization), Jeddah, Saudi Arabia.

Maintaining tissue viability is a challenge of varying degrees during every plastic reconstructive surgery (PRS) procedure. Sir H. Gillies once stated: *“Plastic surgery is a constant battle between blood supply and beauty.”*^1^

Occasionally, a routine procedure may end up with a complication of ischemia and tissue loss. This is seen, for example, in rhinoplasty, with nasal skin viability issues, facelift flap compromise, digital ischemia in a hand surgery, or fat necrosis in lipotransfers. In fact, even a filler injection into the face involves an ischemic element with a rare risk of tissue necrosis. When it comes to specific flaps or free tissue transfers, then the focus on the intactness of blood supply becomes much more obvious. Hence, the concern about tissue viability is always there.

Some tension at the site of closure is a normal expectation in all PRS procedures. Nonetheless, when it is significant, “tension” means an invitation to problems and wound edge ischemia, with accompanying risk of dehiscence *[Tension ∝ Ischemia].* One generally reverts to undermining and advancement (local flap) or other means according to the “reconstructive ladder principle.” This simply means wound tension is inversely proportional to the amount of tissue mobilization, or advancement [*Tension ∝ 1/Tissue transfer].*

Tissue mobilization in attempt to relieve tension could also prove to be potentially detrimental to perfusion when it is miscalculated, depending on the physical factors in locoregional flaps or upon reestablishing the microcirculation in case of free tissue transfers.^2–4^ [*Tissue transfer ∝ schemia*].

**Fig. 1.**
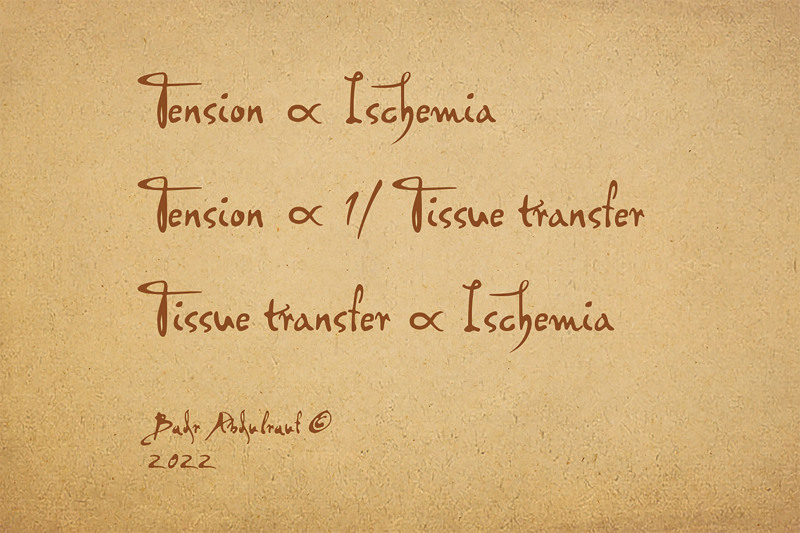
The three golden formulas that govern the field of PRS technically speaking. 1) The degree of Tension at site of closure is directly proportional to extent of wound edge ischemia. 2) Amount of the tissue mobilized is inversely proportional to the wound Tension. 3) More the maneuvers used for reducing tension or reconstructing a defect, the element of ischemia is also associated and relatively increased. Surgical risks are associated with all specialties but in PRS, the interventions are commonly and barely optional. The author felt the above formulas deserved to be portrayed in Leonardo da Vinci’s style.

Nevertheless, free muscle transfer, for instance, is often indicated to improve blood supply of a certain region; however, when a free flap fails, then one is back to square one, besides a donor tissue loss. The original defect to be reconstructed, therefore, often remains like a “*debt to be paid*.” The concept we are conveying here is the fact that any tissue repositioning or a flap procedure does involve a risk to the tissue’s vitality. The three formulas (Fig. 1) are somewhat contradictory to each other mathematically, but this is the reality. We believe that it is essential for students of surgery, particularly PRS, to be acquainted with these.

**Fig. 2.**
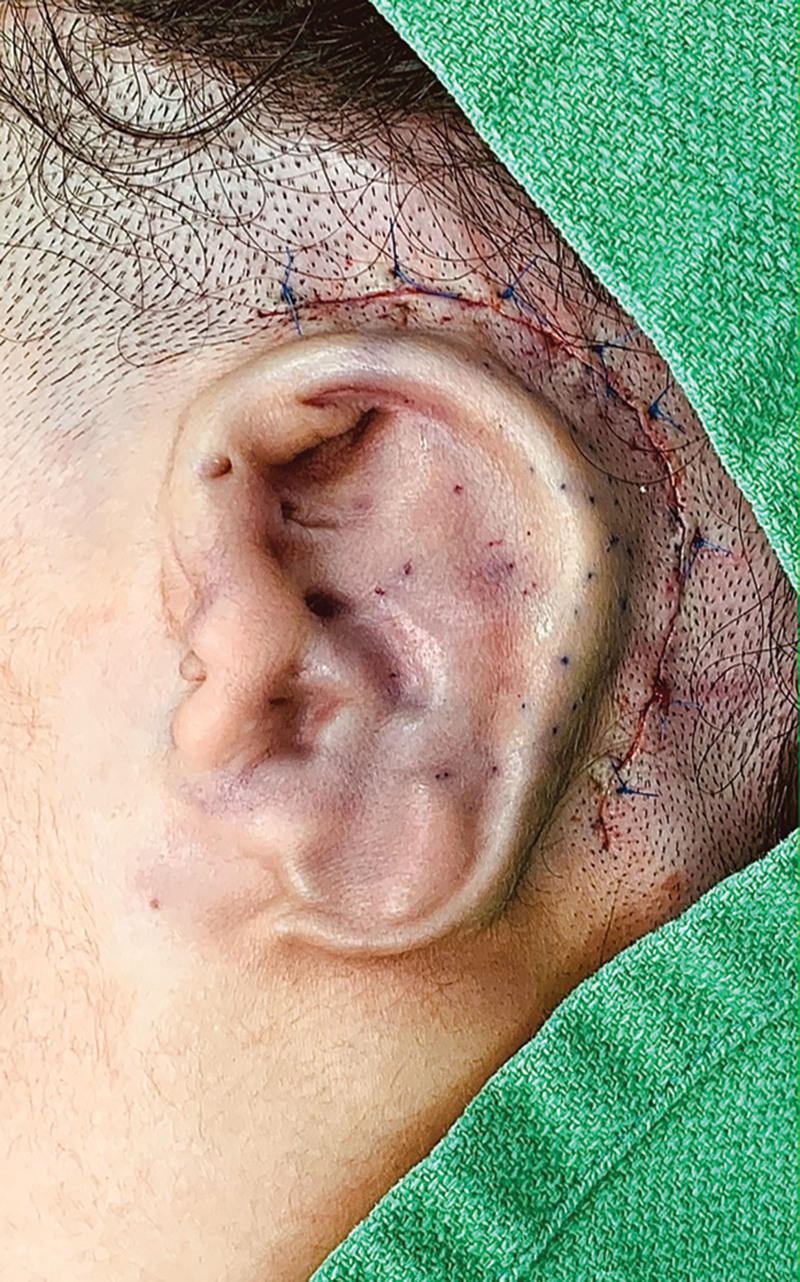
The end of the first stage of microtia reconstruction with autogenous rib cartilage grafts. A compromise of skin flap is a possible risk and often a feared complication, which reflects the “two-edge sword” nature of this procedure and many others. The three formulas in Figure 1 can be perfectly correlated here.

There is often a thin line that exists between obtaining marvelous results and ending up with complications of tissue necrosis and a possible exposure of implants or other structures. Numerous examples exist: various body lift procedures, augmentation mastopexy, free tissue transfers, etc.^5^ To elaborate, an example of a microtia reconstruction can be used. To achieve the best possible shape, the skin flap should be optimally thin, and meanwhile, its adequate perfusion is obviously critical. If more skin is recruited from the surrounding areas, it would reduce tension and accommodate a higher profile detailed cartilaginous framework. A perfect balance needs to be achieved for the reconstruction to work (Fig. 2).

The message from this brief communication is to acknowledge a technical process that characterizes the field of PRS, and to express it in the form of three mathematical formulas.
